# Narrow Scope of Vitamin C Review

**DOI:** 10.1371/journal.pmed.0020308

**Published:** 2005-09-27

**Authors:** William Sardi

**Affiliations:** **1**Knowledge of HealthSan Dimas, CaliforniaUnited States of America

Covering 60 years of research without mentioning a paper that highlights flaws in the literature, in a review, should negate any conclusion. In the Cochrane review by Douglas et al. [1], which is referenced in the Best Practice article by Douglas and Hemilä [2], there was no mention of the revealing paper published last year by Padayatty et al. [3], which shows that three-times greater blood concentration can be achieved with an oral dose of vitamin C than previously thought possible. Since viruses increase the demand for ascorbic acid, the oral doses used in the reviewed studies appear trivial, and would not be expected to produce any positive effect. Compare human oral dose studies to what animals synthesize throughout the day. It is obvious that a single dose of a water-soluble vitamin, regardless of the number of milligrams consumed, will not elevate blood plasma levels enough to produce a preventive or therapeutic effect.
